# How the COVID-19 pandemic affected routine child vaccination: an integrative review

**DOI:** 10.31744/einstein_journal/2025RW1119

**Published:** 2025-03-14

**Authors:** Julia Stoeterau Moré, Daniel Rodrigo Serbena, Luiz Gustavo Gusson de Camargo, Pedro Augusto Clemente, Fernando Sluchensci dos Santos, Juliana Sartori Bonini

**Affiliations:** 1 Laboratório de Neurociências e Comportamento Universidade Estadual do Centro-Oeste Guarapuava PR Brazil Laboratório de Neurociências e Comportamento, Universidade Estadual do Centro-Oeste, Guarapuava, PR, Brazil.

**Keywords:** COVID-19, Pandemics, Vaccines, Vaccination coverage, Vaccination, Immunization, Immunization schedule, Child health, Child, Infant, newborn

## Abstract

**Background:**

Child immunization plays a critical role in preventing numerous diseases. However, the COVID-19) pandemic has profoundly disrupted healthcare systems globally, including routine child vaccination programs.

**Objective:**

To provide an overview of the reduction in vaccine coverage among infants and children during the pandemic and analyze the potential impacts of decreased child immunization during this period.

**Methods:**

A comprehensive search was conducted using the MeSH terms “Child,” “Vaccination”, and “COVID-19,” along with their synonyms. Systematic reviews published between March 11, 2020, and June 1, 2023, in Portuguese or English were included. Databases searched included PubMed, BVS (Biblioteca Virtual em Saúde), Embase, and Scopus. Two blinded independent reviewers performed the selection process, with conflicts resolved by a third reviewer. The AMSTAR-II tool was used to assess the methodological quality of the included studies.

**Results:**

Of the 1,534 eligible articles, only 13 addressed the pandemic’s impact on children’s vaccination coverage. Most studies involved multiple countries and reported a significant decrease in children’s vaccination coverage due to the COVID-19 pandemic.

**Conclusion:**

The findings were heterogeneous but consistently highlighted the substantial impact of the COVID-19 pandemic on routine vaccination coverage in most countries. Further research is needed to explore the epidemiological consequences of disruptions to vaccination schedules, potentially guiding public policies and raising awareness about the importance of adhering to health protection programs.

## INTRODUCTION

Child immunization is a critical practice that prevents numerous diseases and saves an estimated two to three million lives annually, significantly reducing global child mortality and morbidity.^1-2^ This practice is considered one of the most cost-effective health interventions for decreasing disease prevalence. To assess its effectiveness, terms such as “vaccination coverage” or “vaccination uptake” are commonly used. These terms indicate the proportion of children who receive a specific vaccine within a defined timeframe. Adherence to vaccination schedules is crucial to ensuring maximum efficacy against vaccine-preventable diseases and preventing large outbreaks of common illnesses.^3^

However, the COVID-19 pandemic has led to a decline in child vaccination rates. Interruptions in vaccination services were widespread and caused by multiple factors. Even when services remained operational, many individuals were reluctant to visit healthcare facilities due to concerns about potential COVID-19 exposure. Furthermore, transportation and movement restrictions during lockdowns, shortages of healthcare professionals, and inadequate protective equipment further hindered access to vaccination services. An equally significant factor was the closure of schools, which disrupted routine immunization programs typically conducted in school settings.^4^

The first case of COVID-19, caused by severe acute respiratory syndrome coronavirus-2 (SARS-CoV-2), was reported at a seafood market in Wuhan, China, in late 2019. Within weeks, the virus had spread globally, following an exponential growth contamination curve. On March 11, 2020, the World Health Organization (WHO) officially declared COVID-19 a pandemic. As of May 19, there were 473 regions affected, with a total of 458 confirmed COVID-19 cases and 316. In total, 169 confirmed deaths occurred globally.^5-7^

Children account for a minority of SARS-CoV-2 infections, with most infections acquired through contact with adults.^8^ The majority of cases are mild, with severe disease occurring in only 1% of children and a mortality rate of 0.1%.^9^ In the United States (USA), 15,594,079 COVID-19 cases were reported in children, accounting for 17.9% of all COVID-19 cases, as of May 2023. The global incidence rate in children was 20,718 cases per 100,000 children.^10^ Symptoms of COVID-19 in children are generally consistent with those of acute respiratory infections and include fever, cough, sore throat, sneezing, myalgia, and fatigue.^8^

The benefits of the COVID-19 vaccine include a reduction in severe symptoms and mortality, containment of the disease’s spread, and the potential to achieve herd immunity. A common indicator of vaccine safety is any “Adverse Event Following Immunization (AEFI),” which can encompass any unintentional or unfavorable signal, abnormal laboratory results, symptoms, or disease. The COVID-19 vaccine presented an AEFI rate of 10.9% in the BNT162b2 vaccine group compared with 9.2% in the Placebo Group, and the global incidence of AEFI in the BNT162b2 vaccine and Placebo Groups was 6% and 5.9%, respectively.^11^

Non-serious adverse reactions reported in children include pain, swelling, redness at the injection site, fatigue, malaise, headache, myalgia, arthralgia, fever, lymphadenopathy, and chest pain. Furthermore, the most common severe reaction detected during safety monitoring was myopericarditis. Severe reactions to the COVID-19 vaccine are rare.^12^ Therefore, the benefits outweigh the risks of viral contamination.

The COVID-19 pandemic severely impacted healthcare systems, influencing how individuals sought and received care. For example, the pandemic interrupted immunization efforts in ≥68 countries, affecting nearly 80 million children. Globally, it is estimated that due to the COVID-19 pandemic, approximately 23 million children were not immunized in 2020.^13,14^ The most commonly cited reasons by parents for not vaccinating their child against COVID-19 pertain to concerns regarding potential negative long-term effects. Other factors included worries regarding adverse reactions, the belief that COVID-19 is not serious enough for their child to need the vaccine, and the perception that the natural immune system provides sufficient protection against COVID-19.^15^

Furthermore, according to the World Health Organization (WHO), most COVID-19 vaccines are administered in high- and upper-middle-income countries. However, individuals in low- and middle-income countries (LMICs) do not have the same access to vaccines, highlighting a potential vaccine inequality.^16^ When combined with the fact that the developing world is undergoing a demographic transition characterized by a high proportion of children relative to the total population, a significant number of children remain unvaccinated compared to those in the developed world.^17^

## OBJECTIVE

This article aims to comprehend the motives that led to the reduction in routine vaccinations for children and infants during the COVID-19 pandemic and to analyze the potential impacts of this decline in child immunization during this period. Furthermore, we seek to answer the question, “What are the motives and impacts of this reduction?”.

### Methodology

An integrative review is the most comprehensive methodological approach among review methods. This approach allows for the inclusion of both experimental and non-experimental studies, thereby providing a thorough understanding of the phenomenon under analysis. Furthermore, it integrates data from both theoretical and empirical literature, serving various purposes, including defining concepts, reviewing theories and evidence, and analyzing methodological challenges specific to a given topic.^18^

Through systematic reviews, this integrative review aimed to analyze the impact of the COVID-19 pandemic on delays in routine vaccinations for children and infants.

### Eligibility

Systematic reviews examining the impact of the COVID-19 pandemic on the routine vaccination of children and infants’ were selected. Only articles published between 2020 and 2023 were included, as the WHO declared COVID-19 a pandemic on March 11, 2020. There were no restrictions on the language of the articles. Studies that only included adult and elderly populations were excluded.

### Databases and search strategy

Searches were conducted in June 2023 in the databases PubMed, BVS (“*Biblioteca Virtual de Saúde*”), Embase, and Scopus for systematic reviews addressing the impacts of the COVID-19 pandemic. The search utilized the following terms: (Coronavirus OR COVID-19 OR SARS-CoV-2 OR 2019 Novel Coronavirus OR 2019-nCoV OR COVID-19) AND (Vaccination* OR Vaccine* OR Immunization schedule* OR Vaccination schedule* OR Immunization) AND (Child OR Infant* OR Children OR Babies OR Neonates OR Newborn). The search was limited to the titles and abstracts of the articles. A third independent reviewer resolved any discrepancies between the authors.

### Reviews selection

Initially, articles that were previously selected based on the established search criteria were analyzed by two independent researchers who identified eligible systematic reviews according to: the title of the article, the abstract of the articles previously selected by title, and the full text of the articles that were selected based on the abstract. A third independent researcher resolved any discrepancies between the authors.

### Data extraction

A table summarizes the data extraction process. The columns contain information on the names of the authors of the article in question, the year of publication, the aim of the articles, the methodologies used, types of studies conducted, the number of articles reviewed, the countries studied, and the main results obtained.

### Assessment of bias

Each article included in the review was assessed individually by one reviewer using the AMSTAR-II tool. This tool was created for the evaluation of systematic reviews that include randomized or non-randomized studies of healthcare interventions or both.^19^

## RESULTS

**Figure 1:**
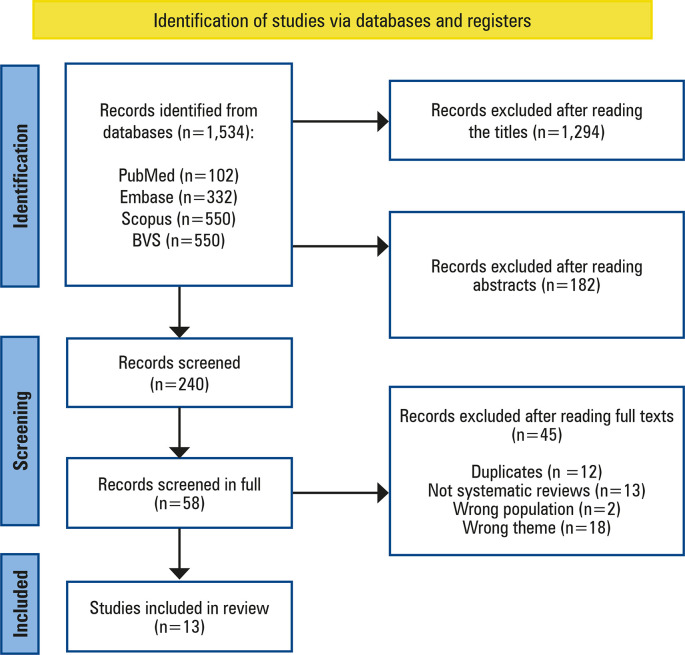
Article selection process

Initially, 1,534 articles were identified in the four databases using the search strategy: 102 from PubMed, 332 from Embase, 550 from Scopus, and 550 from BVS. A total of 1,294 articles were excluded after reviewing the titles and 182 after reviewing abstracts, leaving 58 articles for further evaluation. Of these, 12 were excluded due to being duplicates, 13 because they were not systematic reviews, 2 for not meeting the eligibility criteria regarding the population, and 18 because they addressed topics that differed from the study’s proposal, resulting in the exclusion of 45 articles. Consequently, 13 articles were selected for final analysis. Figure 1 illustrates the study selection process.

*Consider, if feasible to do so, reporting the number of records identified from each database or register searched (rather than the total number across all databases/registers); **If automation tools were used, indicate how many records were excluded by a human and how many were excluded by automation tools.

As stated previously, AMSTAR-II was used to assess the methodological quality of the included studies, comprising 16 questions, seven of which were considered critical and contributed to the overall score of the study.

**Figure 2:**
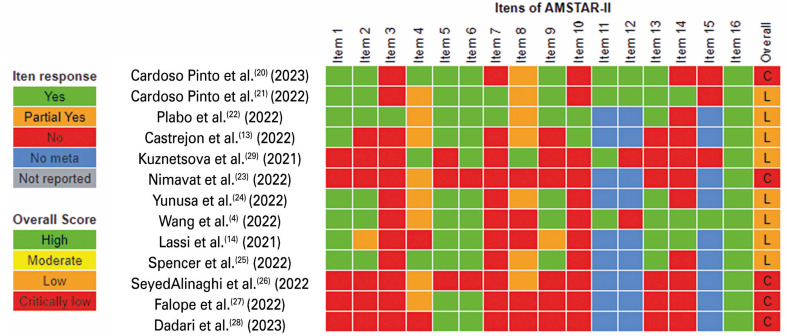
Methodological quality of studies according to AMSTAR-II application

Systematic reviews can be rated as high, moderate, low, or critically low based on their quality. A “high” rating indicates that there are no or only one non-critical weakness, signifying that the review provides an accurate and comprehensive summary of the relevant studies. A “moderate” rating indicates more than one non-critical weakness but no critical flaws, still offering a generally accurate summary. A “low” rating involves one critical flaw that may affect the accuracy of the review. A “critically low” rating indicates the presence of more than one critical flaw. A critical flaw signifies that a critical question was answered “no,” whereas non-critical flaws are related to non-critical questions. The essential questions are identified as “2, 4, 7, 9, 11, 13 and 15.” The results of this evaluation are presented in figure 2.

### Included article characteristics

The selected articles were published between 2020 and 2023, with 2 published in 2021, 10 in 2022, and 2 in 2023. All studies were systematic reviews conducted across several countries. Table 1 provides a summary of the authors, year of publication, objectives, methodology, types of studies, research locations, and results.

**Table 1:** Characteristics of the selected article

Authors	Objective	Methodology	Type of study and number of articles	Regions studied	Results
Cardoso Pinto et al.^(20)^	Summarize the reasons for interruptions in routine childhood immunizations in LMICs during the COVID-19 pandemic	A systematic review (PROSPERO CRD42021286 386) was carried out following the PRISMA 2020 guidelines	A total of thirteen studies were included describing the reasons behind the interruptions: 7 cross-sectional (quantitative), 5 qualitative and 1 mixed methods	LMICs In the cross-sectional studies, the WHO world region with the most respondents was Southeast Asia (49.9%). In qualitative studies, the majority of respondents were from the WHO African region (53.3%) or the Southeast Asia region (44.6%)	Seventeen reasons for interruptions were identified. The most common reasons for interruptions were parental fear of COVID-19 and avoidance of health services
Cardoso Pinto et al.^(21)^	Quantify the levels of interruption of routine immunization in LMICs	A systematic review (PROSPERO CRD42021286 386) of MEDLINE, Embase, Global Health, CINAHL and Scopus was conducted	A total of 39 cross-sectional studies were identified	The studies covered 6 WHO regions unevenly, with Africa (53.8%) being the most common	There was a decline in routine pediatric vaccination, higher in MICs and for vaccines administered at birth
Palo et al.^(22)^	To study the use of maternal and child health (MCH) services during pandemics (Zika, Ebola and COVID-19) and the effectiveness of various interventions carried out to ensure the use of MCH services	A systematic and comprehensive search was carried out on MEDLINE/PubMed, Cochrane CENTRAL, Embase, Epistemonikos ScienceDirect, and Google Scholar	Out of 5.643 citations, 60 potential studies were finally included for analysis, 11 were qualitative, 8 were mixed methods, and 41 were quantitative	The studies included were from high-income countries (n = 23), LMICs (n = 34), and both (n = 3). Geographically, the included studies were widely distributed throughout the world	The findings suggest that during pandemics, the use of MCH care is often affected. Many innovative interventions have been adopted to ensure MCH services
Castrejon et al.^(13)^	Evaluate vaccination coverage data and identify recovery strategies for lost vaccines in selected Latin American countries (Argentina, Brazil, Chile, Colombia, Mexico, and Peru)	A systematic literature review of published articles was carried out to identify vaccination recovery strategies that were published	Of the 696 studies identified, 14 were included in this review	Argentina, Brazil, Chile, Colombia, Mexico and Peru	Overall coverage decreased to varying degrees among the countries investigated. This trend was observed before 2020, suggesting multifactorial reasons for the decline in vaccination rates in Latin America
Nimavat et al.^(23)^	Understanding the challenges faced by India’s health system during a pandemic	The literature search for this review was carried out using PubMed, EMBASE, Scopus, Web of Science and Google Scholar	42 articles	India	The Indian health system was suffering even before the pandemic. The pandemic has stretched health services in India
Yunusa et al.^(24)^	Understanding the international impact of the COVID-19 pandemic on routine vaccination of pregnant women and children aged 0-5 years	The authors conducted a systematic review of quantitative and mixed-methods studies exploring changes in vaccination coverage, vaccination services and vaccine confidence since the start of the COVID-19 pandemic	Thirty studies were included in the review; 2 studies were classified as descriptive analysis studies, 1 as an interim analysis, 8 as observational studies (including ecological, cross-sectional and cohort studies), and 2 as mixed-methods studies (1 descriptive and 1 cross-sectional analysis). 16 studies did not specify their type of study and therefore categorized as observational studies	Twenty studies focused on HICs (Japan, USA, Netherlands, Singapore, Canada, England, South Korea, Sweden, and Italy), and 7 focused on LMICs	Both groups experienced declines in vaccination coverage (up to - 79%), with greater disruptions in the accessibility and delivery of delivery of vaccination services reported within LMICs compared to HICs. The COVID-19 pandemic has resulted in a decrease in vaccination coverage and a reduction in vaccination routine services for pregnant women and babies
Wang et al.^(4)^	Systematically research and summarize the evidence from the literature reporting parents’ willingness/hesitation about vaccinating their children at the time of COVID-19 (in terms of childhood/routine vaccination, seasonal flu vaccination, human papillomavirus (HPV) vaccination and pneumococcal conjugate vaccination (PCV)	Academic articles were identified by searching the electronic databases PubMed, Web of Science (including SSCI and A&HI), EBSCOhost (including CINAL with Full Text, ERIC, MEDLINE, APA PsycArticles and APA PsycINFO) and Scopus (including published articles and pre-print services), covering the published periods of 1966-2022	A total of 20 eligible studies published from 2020-2022 were included for systematic summary by a thematic review, among which 12 studies were included in a meta-analysis conducted with R-.4.2.1	12 countries, including the United States, China, Saudi Arabia, Turkey, Switzerland, Indonesia, Mozambique, Albania, Canada, Israel, Japan and Spain	Based on the evidence provided by this review, it is necessary to design and implement parental intervention programs for the promotion of childhood vaccination targeting the specific local context/circumstances in different countries/regions
Lassi et al.^(14)^	Assess the impact on vaccine coverage worldwide and identify potential underlying factors	A systematic search strategy was employed in the PubMed, Embase, BioRxiv and COVID-19 databases of the WHO from December 2019 to September 15, 2020	A total of 17 observational studies were included	Of the 17 observational studies, 10 explored immunization campaigns in high-income countries (HICs) and the remaining 7 in LMICs. Of the HIC studies, 5 were carried out in the United States, 2 in the United Kingdom and 1 each in France, Italy and Spain. Of the LMICs, 4 were from Pakistan, 1 study was carried out in 2 countries, namely Afghanistan and Pakistan, and 1 was from South Africa and Sierra Leone	The results suggest that there has been a reduction in vaccination coverage and a decline in the number of vaccines administered, which led to children losing their vaccine doses
Spencer et al.^(25)^	Assess evidence and evaluate the impact of the COVID-19 pandemic on inequalities in routine childhood vaccination coverage	The search strategy for published and pre-printed literature was conducted by a health sciences librarian at the University of Warwick Library (SJ) to identify published studies that reported data on inequality in routine childhood vaccination coverage during the COVID-19 pandemic. The review protocol was registered with PROSPERO (CRD 42021257431)	91 out of 1453 were selected for complete revision, and 13 fit into the selection criteria	The study did not specify the regions included. Only stated HICs and LMICs	The narrative synthesis found moderate evidence of inequality in the reduction of vaccination coverage of children during the COVID-19 lockdowns and moderately strong evidence of an increase in inequality compared to the pre-pandemic months (before March 2020)
SeyedAlinaghhi et al.^(26)^	To systematically review studies that addressed the impacts of the COVID-19 pandemic on vaccination coverage in children and adolescents worldwide	Systematic search for relevant studies using the keywords in the PubMed, Web of Science and Cochrane databases	26 eligible studies	USA, Saudi Arabia, Japan, Brazil, Ethiopia, the Republic of Korea, Singapore, China, Morocco and Italy	Twenty-one studies showed a decrease in vaccination rates in children during the COVID-19 pandemic, whereas three studies found an increase or no significant change in vaccination only against influenza
Falope et al.^(27)^	Describe studies that illustrate elements of health system resilience that have improved the ability of specific immunization programs to provide routine immunization services during the pandemic	A systematic search was carried out in Embase, Web of Science, PsychInfo and the gray literature between January 1, 2020, and November 12, 2021	The review of the full text led to the selection of 34 studies, which were included in the final review process	34 studies comprising 11 publications that included data from Asia, 10 that included data from America, 9 that included data from Europe, 5 that included data from South America and 4 publications that included data from Oceania	The studies indicated that there were immunization programs in all regions that demonstrated program resilience by using tools to mitigate the impact of the COVID-19 pandemic on routine immunizations
Dadari et al.^(28)^	Summarize the routine setback in immunization after the COVID-19 pandemic and predictors of coverage and identify pro-equity strategies in urban and peri-urban settings through a systematic search of the published literature	Two databases, PubMed and Web of Science, were exhaustively searched using search terms and synonyms, resulting in 608 peer-reviewed articles identified	Based on the inclusion criteria, 15 articles were included in the final review	A total of 20 countries with the highest number of children who have received a zero dose and are home to >75% of these children in 2021 were prioritized	Several studies have clearly documented a decline in coverage in urban and peri-urban areas, with some predictors or challenges for optimal coverage, as well as some pro-equity strategies implemented or recommended in these studies
Kuznetsova et al.^(29)^	Evaluate child vaccination programs implemented in selected countries	The review was carried out following the guidance issued by the Preferred Reporting Items for Systematic Reviews and Meta-Analyses (PRISMA) and the Cochrane Handbook for Systematic Reviews of Interventions, including its technical supplements	A total of 466 full-text articles were assessed for eligibility, and 26 articles from seven countries were included in the synthesis	The review included studies from 7 countries: 7 studies on Italy, 4 on Germany, 3 on France, 4 on Latvia, 3 on Serbia, 3 on Moldova and 2 on Ukraine	Vaccination coverage for almost all vaccines improved significantly in Ukraine after the implementation of the mandate, despite the COVID-19 pandemic

The COVID-19 pandemic has led to significant disruptions in vaccination coverage worldwide, as both high- and low- and middle-income countries face challenges.^25^ In high-income countries (HICs) such as the USA, 61.7% of physicians reduced office hours,^24^ whereas European countries, including Germany, France, and Spain, experienced notable declines in vaccine delivery, particularly for Measles, Mumps and Rubella vaccine (MMR) and HPV.^14,29^ Conversely, Ukraine and South Korea maintained or even increased their vaccination rates through the use of technology and public health measures.^29^ In contrast, Sweden has faced growing parental concerns regarding infant vaccinations.^24^

Among LMICs, countries such as Argentina, Brazil,^13^ and India^23^ have experienced significant declines in vaccination coverage. India, in particular, has seen an estimated 27 million children missing vaccinations, resulting in a 40% increase in mortality rates. Africa has demonstrated mixed outcomes in vaccine coverage,^20,21^ whereas in Southeast Asia and the Western Pacific, approximately 80% of both public and private vaccinations have been disrupted.^21^ The most common barriers to vaccine access include fear of COVID-19 infection, transportation challenges, and logistical issues such as shortages of staff and equipment. Rural areas in both HICs and LMICs have been more adversely affected than urban regions, and private healthcare services have generally been more affected than public ones. Low- and middleincome countries have encountered vaccine supply issues, whereas, in HICs, the transition to virtual consultations and fear of COVID-19 have contributed to decreased vaccine uptake.

Disparities in healthcare access were more pronounced among various social strata and sectors across both country categories. Table 2 presents the primary differences in vaccine coverage, causes, and other considerations between HICs and LMICs.

**Table 2:** Comparison between high income countries and low and middle income countries

	High-income countries	Low-income countries
Vaccine coverage	- The pandemic caused a reduction in operation hours and increased the duration of consultations. As an example, across the US, 61.7% of physicians offered reduced office hours for in-person visitations^(21)^ - Germany^(29)^ showed a 7-fold decrease in measles cases, a 2.5-fold decrease in rubella cases, a 3-fold decrease in pertussis cases, a 2-fold decrease in Hib diseases, and a 2-fold decrease in varicella, which may be due to a mandatory vaccination law - In France,^(14,29)^ all mandatory priming doses and booster dispensations were reduced, especially in the first 4 weeks of the initial lockdown. Furthermore, along with Spain, it suffered major declines in vaccine deliveries, including MMR and HPV vaccines. England also showed a significant loss, but lower when compared to the first 2 countries - In Ukraine,^(29)^ there was a significant increase in vaccine coverage in 2020 compared to 2018 across Polio, DTP, HepB and Hib vaccines. Furthermore, in comparing 2020 to 2019, the incidence of measles fell by 215 times, mumps by 2.5 times, and rubella by 3.6 times - In Sweden^(24)^, a survey showed a significant portion of parents with increasing concerns surrounding the vaccination of their infant following the pandemic - South Korea^(24)^ maintained high vaccination rates during the COVID-19 pandemic through effective use of technology, including a national immunization registry and smartphone apps for scheduling and reminders. Its strong public health infrastructure, collaboration between private and public sectors, and innovative solutions like drive-through clinics ensured broad access to vaccines	- Median decline of −10.8% across all countries. Upper-middle-income countries and lower-MICs showed greater declines than low-income countries.^(21)^ - Declines during the first 3 months of the pandemic were greater than during the remainder of 2020^(21)^ - Africa^(20,21)^ presented mixed results across countries for DTP3 and MCV1 vaccination coverage - In Argentina, Brazil, Chile, Colombia, Mexico and Peru,^(13)^ the change in vaccination coverage worsened in several vaccines (RV, MCV, PCV, pentavalent, BCG) - In India,^(23)^ it was estimated that about 27 million children missed diphtheria tetanus pertussis, which resulted in a 40% increase in mortality in 2021 - Within Brazil,^(28)^ Some of the states with substantial decline rates included Paraná (49.97%), São Paulo (43.25%), and regions such as the North (34.71%), Midwest (21.72%), South (63.50%), and Southeast (34.42%) - In Southeast Asia^(20)^ and the Western Pacific region, 79% of public sector vaccinations were disrupted, and 83% within the private sector
Particular reasons	- The most common reasons were fear of COVID-19 infection, transport challenges faced by both patient and healthcare workers, movement restrictions by lockdown, reduced operating hours and longer consultation times, cancellations and postponements of vaccinations appointments, booking appointments, unawareness of vaccination program’s continuation by parents, logistical problems including staff and equipment shortages, vaccine supply chain issues - Although quantitative studies attributed a bigger share of the blame to reduced healthcare-seeking, as opposed to healthcare-delivery issues, qualitative studies showcased an equal portion of the blame - Furthermore, in LMICs, barriers were mostly skewed towards vaccine inadequacy, vaccine hesitancy, and calling-off clinics. In HICs, fear of contracting COVID-19 and changes in management norms like shifting towards virtual consultations attributed to a decline in vaccine uptake	
Other considerations	- In both categories of countries, rural areas suffered a higher drop in healthcare visits than urban areas - Studies agreed on an international inequity in pandemic effects on HIC and LMIC; however, these diverge when comparing different social strata classes - Both categories had the private sector as most affected when compared to the public one - Decreases in vaccine administrations between public and private sectors were primarily seen within HICs, whereas in LMICs, these differences in vaccine administrations by setting were typically reported between fixed and outreach services	

HICs: high-income countries; LMICs: low- and middleincome countries.

## DISCUSSION

### Coverage impact

The COVID-19 pandemic has affected nearly every country worldwide, including significant disruptions to immunization programs. A noticeable decline in vaccination coverage has been observed globally, with approximately 25 million individuals under-vaccinated in 2021. Of these, 18 million did not receive the first dose of the diphtheria-tetanus-pertussis vaccine (DTP).^28^ A study observed a reduction in compliance with vaccinations, particularly for BCG, Pentavalent, Polio and Measles.^22^ Both HICs and LMICs experienced reduced vaccination coverage and difficulties with immunization services. In some instances, the changes in LMICS and HICS were similar; however, due to these challenges and low pre-existing vaccination rates in LMICs, their post-pandemic vaccine coverage rates were lower than those of HICs, indicating that the pandemic was a greater concern for this group.^14,24^

A cross-sectional study reported a median decline of >10% in routine childhood vaccinations in LMICs, with the majority of countries represented in the analysis from the WHO African region. This study demonstrated that the most significant decline in child immunization occurred during the first three months of 2020, supporting the idea of recovery, although declines persist.^21^

Additionally, another systematic review analyzed both cross-sectional (quantitative) and qualitative studies from LMICs and also found a decrease in vaccination coverage.^20^

A study aimed at understanding the challenges faced by the Indian healthcare system during the pandemic revealed a significant reduction in childhood vaccination schedules. The evolution of the pandemic in India initially resulted in a complete shutdown of all child vaccination programs due to a major lockdown. During this period, an estimated 27 million children missed the tetanus-diphtheria-pertussis vaccine, resulting in a 40% increase in mortality over the following year.^23^

An article that sought to quantify routine baby immunization reductions during the COVID-19 pandemic in Ecuador observed that the pandemic significantly impacted child immunization programs across all national territories. The same study also indicated that immunization suffered a grave impact in the “Costas” and “Terras Altas” regions of Ecuador.^30^

Another study aimed to evaluate vaccination coverage data and identify recovery strategies for vaccines missed during the pandemic in selected Latin American countries, including Argentina, Brazil, Chile, Colombia, Mexico, and Peru. The study reported a general decrease in vaccination coverage rates across the region. It also analyzed regional variations in immunization, indicating that factors such as regional and cultural differences, levels of concern about infection, declining birth rates, and limited access to healthcare centers may have contributed to these disparities. Notably, Brazil experienced a slight increase in immunization rates against DTP increased during the pandemic. Similarly, Mexico reported higher vaccination coverage rates for most vaccines, with the exception of the BCG vaccine.^13^

However, some systematic reviews have reported studies indicating little interference from the COVID-19 pandemic on routine childhood vaccination coverage in some HICs. For example, one study showed that in Ukraine, COVID-19 did not cause any major interruptions in childhood vaccination coverage.^29^ It is worth noting that this same article also showed that the pandemic negatively affected vaccination coverage in France. In France’s first 10 months of the 2020 COVID-19 pandemic, all mandatory priming and booster dose dispensations were reduced compared to the expected estimates based on the previous year. The reduction was particularly impressive during the first 4 weeks of the first lockdown, especially for the MMR. During the immediate post-lockdown period, the counts of all mandatory vaccine dispensers remained lower than expected.^29^ Another systematic review indicated that some countries managed to sustain their childhood immunization programs through catch-up strategies, facing only brief periods of decline in immunization rates, such as the Republic of Korea, Switzerland, and Sweden.^26^

### Reasons for coverage drop

The causes of the decrease in child vaccination have been multifactorial, as indicated by many studies. Among the causes listed are: fear of contracting COVID-19 and fear of children contracting COVID-19, particularly in healthcare settings; lockdown policies encouraging families to stay at home; mobility restrictions; reduced transportation; disruptions in the provision of health services and the closure of immunization centers; challenges with vaccine supply; scheduling difficulties; lack of professionals; vaccine hesitancy; lack of family support.^20-22^

Another study also examined the differences in the main causes of decreased immunization between LMICs and HICs. In the former, the barriers were mainly related to vaccine inadequacy, hesitancy, and withdrawal from clinics. In the latter, the fear of contracting COVID-19 and changes in management norms, such as switching to virtual consultations, were attributed to a reduction in the vaccination rate.^22^

### Mitigation approaches

Despite the difficulties posed by the pandemic, a range of strategies have been deployed with the objective of guaranteeing comprehensive vaccination coverage. In India, a considerable number of parents have elected to utilize private healthcare facilities for their children’s vaccinations, largely because of concerns about infection. This has resulted in some facilities reducing waiting periods and modifying schedules to minimize the spread of the virus. A study conducted in Ethiopia revealed that existing immunization services in urban areas were insufficient. The study proposes a series of solutions, including the expansion of outreach to marginalized populations, the strengthening of public-private partnerships, the engagement of private health facilities, and the integration of digital tools such as mHealth reminders. A study conducted in urban slums in Nigeria revealed that training older women through participatory learning improved their knowledge and advocacy for infant vaccination. Furthermore, in Oromia, recommendations for enhancing MCV2 uptake include reducing vaccination waiting times, enhancing awareness among caregivers, and focusing on older mothers.^28^

### Consequences

Furthermore, owing to the inequality in the distribution of the COVID-19 vaccine between HICs and LMICs, the former presented faster post-pandemic recovery than the latter, which received fewer COVID-19 vaccines.^31^ This inequality also means that LMICs will have a slower economic recovery, explained by the positive relationship between the share of vaccinated individuals and GDP, and consequently further hamper routine vaccination, increasing the most vulnerable and in most need of assistance areas’ health fragility and the possibility of death by preventable diseases.^32^

Declines in routine vaccination have raised concerns about future morbidity and mortality rates due to diseases that can be prevented through immunization.^23^ This decrease in vaccination rates could result in the global spread of diseases^21^ and the occurrence of major public health threats such as polio and measles. A four-fold increase has also been observed in polio cases in polio-endemic countries. The resurgence of polio in previously polio-free countries has increased, and the discontinuation of polio immunization programs may provide the virus with an environment conducive to its spread, potentially resulting in the worldwide export of infections. As measles is a highly infectious disease, a small decrease in routine measles vaccinations could lead to large and explosive outbreaks, which could significantly increase child mortality.^14^

To prevent such situations from occurring, it is necessary to improve the resilience of immunization programs, a measure implemented during adversity, such as an epidemic or pandemic, to maintain the stability and sustainability of the essential functions of health systems. To this end, strategies can be included, such as prioritizing vaccination campaigns, making adequate funding available for immunization, ensuring system readiness for new vaccines, noticing and quickly addressing hesitancy, and promoting confidence in all aspects of immunization, including education. These actions are key to achieving and maintaining a good vaccination coverage rate and preventing and recovering from disease outbreaks.^27^

## CONCLUSION

This study aimed to analyze the potential reasons associated with the decline in childhood vaccination rates during the pandemic. The findings are heterogeneous; however, it is notable that the COVID-19 pandemic significantly impacted routine vaccinations and coverage in most of the countries analyzed, especially in low- and middle-income countries. This can be attributed to several distinct factors. In light of this scenario, the authors believe that further research is necessary to investigate the repercussions of disruptions in the vaccination schedule from an epidemiological perspective. This research may stimulate the development of public policies aimed at addressing and raising awareness regarding the importance of adherence to specific health protection programs, such as vaccination.

## References

[1] Balgovind P, Mohammadnezhad M (2022). Factors affecting childhood immunization: thematic analysis of parents and healthcare workers' perceptions. Hum Vaccin Immunother.

[2] Kaufman J, Ryan R, Walsh L, Horey D, Leask J, Robinson P (2018). Face-to-face interventions for informing or educating parents about early childhood vaccination. Cochrane Database Syst Rev.

[3] Hadjipanayis A (2019). Compliance with vaccination schedules. Hum Vaccin Immunother.

[4] Wang Z, Chen S, Fang Y (2022). Parental Willingness and Associated Factors of Pediatric Vaccination in the Era of COVID-19 Pandemic: A Systematic Review and Meta-Analysis. Vaccines (Basel).

[5] World Health Organization (WHO) (2020). Coronavirus disease (COVID-2019) situation reports.

[6] Shokoohi M, Osooli M, Stranges S (2020). COVID-19 Pandemic: What Can the West Learn From the East?. Int J Health Policy Manag.

[7] Mazinani M, Rude BJ (2021). The novel zoonotic Coronavirus disease 2019 (COVID-19) pandemic: health perspective on the outbreak. J Healthc Qual Res.

[8] Howard-Jones AR, Bowen AC, Danchin M, Koirala A, Sharma K, Yeoh DK (2022). COVID-19 in children: I. Epidemiology, prevention and indirect impacts. J Paediatr Child Health.

[9] Chaiyakulsil C, Sritipsukho P, Satdhabudha A, Bunjoungmanee P, Tangsathapornpong A, Sinlapamongkolkul P (2022). An epidemiological study of pediatric COVID-19 in the era of the variant of concern. PLoS One.

[10] American Academy of Pediatric (AAP) (2020). Children and COVID-19: State Data ReportA joint report from the American Academy of Pediatrics and the Children's Hospital Association summary of publicly reported data from 49 states, NYC, DC, PR, and GU.

[11] Tian F, Yang R, Chen Z (2022). Safety and efficacy of COVID-19 vaccines in children and adolescents: A systematic review of randomized controlled trials. J Med Virol.

[12] Morello R, Pepe M, Martino L, Lazzareschi I, Chiaretti A, Gatto A (2022). COVID-19 review shows that benefits of vaccinating children and adolescents appear to outweigh risks of post-vaccination myopericarditis. Acta Paediatr.

[13] Castrejon MM, Leal I, de Jesus Pereira Pinto T, Guzmán-Holst A (2022). The impact of COVID-19 and catch-up strategies on routine childhood vaccine coverage trends in Latin America: a systematic literature review and database analysis. Hum Vaccin Immunother.

[14] Lassi ZS, Naseem R, Salam RA, Siddiqui F, Das JK (2021). The Impact of the COVID-19 Pandemic on Immunization Campaigns and Programs: A Systematic Review. Int J Environ Res Public Health.

[15] Byrne A, Thompson LA, Filipp SL, Ryan K (2022). COVID-19 vaccine perceptions and hesitancy amongst parents of school-aged children during the pediatric vaccine rollout. Vaccine.

[16] Ning C, Wang H, Wu J, Chen Q, Pei H, Gao H (2022). The COVID-19 Vaccination and Vaccine Inequity Worldwide: An Empirical Study Based on Global Data. Int J Environ Res Public Health.

[17] Nieves JJ, Stevens FR, Gaughan AE, Linard C, Sorichetta A, Hornby G (2017). Examining the correlates and drivers of human population distributions across low- and middle-income countries. J R Soc Interface.

[18] Souza MT, Silva MD, Carvalho R (2010). Integrative review: what is it? How to do it?. einstein (Sao Paulo).

[19] Shea BJ, Reeves BC, Wells G, Thuku M, Hamel C, Moran J (2017). AMSTAR 2: a critical appraisal tool for systematic reviews that include randomised or non-randomised studies of healthcare interventions, or both. BMJ.

[20] Cardoso Pinto AM, Shariq S, Ranasinghe L, Sundar Budhathoki S, Skirrow H, Whittaker E (2023). Reasons for reductions in routine childhood immunisation uptake during the COVID-19 pandemic in low- and middle-income countries: a systematic review. PLOS Glob Public Health.

[21] Cardoso Pinto AM, Ranasinghe L, Dodd PJ, Budhathoki SS, Seddon JA, Whittaker E (2022). Disruptions to routine childhood vaccinations in low- and middle-income countries during the COVID-19 pandemic: a systematic review. Front Pediatr.

[22] Palo SK, Dubey S, Negi S, Sahay MR, Patel K, Swain S (2022). Effective interventions to ensure MCH (Maternal and Child Health) services during pandemic related health emergencies (Zika, Ebola, and COVID-19): a systematic review. PLoS One.

[23] Nimavat N, Hasan MM, Charmode S, Mandala G, Parmar GR, Bhangu R (2022). COVID-19 pandemic effects on the distribution of healthcare services in India: a systematic review. World J Virol.

[24] Yunusa A, Cabral C, Anderson E (2022). The impact of the Covid-19 pandemic on the uptake of routine maternal and infant vaccines globally: A systematic review. PLOS Glob Public Health.

[25] Spencer N, Markham W, Johnson S, Arpin E, Nathawad R, Gunnlaugsson G (2022). The Impact of COVID-19 pandemic on inequity in routine childhood vaccination coverage: a systematic review. Vaccines (Basel).

[26] SeyedAlinaghi S, Karimi A, Mojdeganlou H, Alilou S, Mirghaderi SP, Noori T (2022). Impact of COVID-19 pandemic on routine vaccination coverage of children and adolescents: a systematic review. Health Sci Rep.

[27] Falope O, Nyaku MK, O'Rourke C, Hermany LV, Plavchak B, Mauskopf J (2022). Resilience learning from the COVID-19 pandemic and its relevance for routine immunization programs. Expert Rev Vaccines.

[28] Dadari I, Belt RV, Iyengar A, Ray A, Hossain I, Ali D, Danielsson N, Sodha SV, The Global Urban Immunization Working Group (2023). Achieving the IA2030 Coverage and Equity Goals through a Renewed Focus on Urban Immunization. Vaccines (Basel).

[29] Kuznetsova L, Cortassa G, Trilla A (2021). Effectiveness of Mandatory and Incentive-Based Routine Childhood Immunization Programs in Europe: a Systematic Review of the Literature. Vaccines (Basel).

[30] Suárez-Rodríguez GL, Salazar-Loor J, Rivas-Condo J, Rodríguez-Morales AJ, Navarro JC, Ramírez-Iglesias JR (2022). Routine Immunization Programs for Children during the COVID-19 Pandemic in Ecuador, 2020-Hidden Effects, Predictable Consequences. Vaccines (Basel).

[31] Suárez-Álvarez A, López-Menéndez AJ (2022). Is COVID-19 vaccine inequality undermining the recovery from the COVID-19 pandemic?. J Glob Health.

[32] Gozzi N, Chinazzi M, Dean NE, Longini IM, Halloran ME, Perra N (2023). Estimating the impact of COVID-19 vaccine inequities: a modeling study. Nat Commun.

